# Having a voice and saving lives: a qualitative survey on employment impacts of people with lived experience of drug use working in harm reduction

**DOI:** 10.1186/s12954-020-00453-5

**Published:** 2021-01-06

**Authors:** People with Lived Expertise of Drug Use National Working Group, Brandi Abele, Jennifer Bowser, Loretta Brown, Julien Carette, Frank Crichlow, Alexandra de Kiewit, Hugh Lampkin, Dawn Lavand, Sean LeBlanc, Alex Sherstobitoff, Rick Sproule, Natasha Touesnard, Karen Turner, Dean Wilson, Kelsey Van Pelt, Tamar Austin, Jade Boyd

**Affiliations:** 1 British Columbia Centre on Substance Use, 400 - 1045 Howe Street, Vancouver, BC V6Z 2A9 Canada; 2 Department of Medicine, University of British Columbia, St. Paul’s Hospital, 608-1081 Burrard Street, Vancouver, BC V6Z 1Y6 Canada

**Keywords:** Canada, Drugs, Stigma, Harm reduction, Overdose, Peer employment

## Abstract

**Background:**

Ongoing legal and social discrimination, and stigmatization of people with lived experience of drug use (PWLE) continues to contribute to overdose-related deaths in Canada. The involvement of PWLE working in harm reduction services has proven effective in decreasing drug-related harms among PWLE; however, there exist unintended negative impacts. PWLE working in harm reduction services risk overextending themselves beyond employment parameters (e.g., emotional labor) with few systems in place (e.g., employment advocacy) for support. While meaningful participation of PWLE in harm reduction programs is critical to addressing the overdose crisis, their labor in Canada’s overdose response commands further investigation and recognition. This paper examines some of the benefits and negative aspects of working in harm reduction among PWLE.

**Methods:**

Fifty qualitative surveys were completed by PWLE working in harm reduction services from across Canada at the National 2018 Stimulus conference held in Edmonton, Alberta. The surveys focused on the benefits and negatives of ‘peer’ employment and recommendations for organizational transformation through short answer written sections. Surveys were analyzed thematically using NVivo, informed by critical perspectives on substance use, with attention to key re-occurring themes on employment equity.

**Results:**

While participants described multiple benefits of working in harm reduction services, such as the valuing of their expertise by fellow ‘peers,’ growing skill sets, countering stigma, and preventing overdose deaths, issues of workplace equity were significantly identified. Stigma, tokenism, workplace discrimination, including power and pay inequities, as well as lack of worker compensation and benefits were identified as key factors persisting in the everyday experiences of participants.

**Conclusion:**

Continued exposure to stigma, workplace discrimination, and/or power imbalances, combined with the impact of high stakes employment (e.g., dealing with overdose deaths), can have significant consequences for PWLE working in harm reduction, including burn out. Policy recommendations include large-scale structural changes that address inequities of hierarchical ‘peer’ employment for PWLE, including increased leadership roles for diverse PWLE, pay equity and benefits, unionization, as well as more supportive working environments attentive to the intersecting social-structural factors (poverty, criminalization, racism, gendered violence) impacting the everyday lives of PWLE working in harm reduction.

## Introduction

In response to limitations of conventional drug services, since the 1990s peer-based models of care have become a significant and expanding component of harm reduction interventions in Canada [1–3], including overdose prevention and addiction treatment programs. This follows a large body of evidence indicating that the involvement of people who use(d) drugs extends the reach and *effectiveness* of interventions, including by reaching those who do not access traditional public health programs [4–9]. Within drug-related social and health-based service provision, peers are defined as people with lived experience of drug use (PWLE), who work in harm reduction services and organizations. However, in the field of harm reduction, the term ‘peer’ has also been used to demarcate an invisible boundary that results in material inequities between harm reduction professionals who use(d) drugs and those not defined by their substance use [3]. Here, we draw upon findings from a recent study by and with community harm reduction mobilizers from across Canada who use(d) drugs, highlighting some of the experiences of those impacted by these invisible boundaries. Interrogating ‘peer’ work and its impacts is significant given the magnitude of preventable drug overdose deaths driven by a poisoned illicit drug supply in Canada [10] and the US [11], and the increased uptake of people with lived experience of drug use working in overdose prevention sites and harm reduction services [12, 13].

Since the early 1900s, people who use illicit drugs have experienced legal and social discrimination, stigma, and marginalization. Critical and feminist drug research has drawn attention to how drug policies are gendered, racialized, and class-based; meaning, the impact of drug prohibition plays out differently for diverse men and women, transgender, non-binary and two-spirit peoples [14–18]. The consequences of illicit drug use are directly associated with one’s social status. Therefore, poor, Indigenous, Black, and other racialized people who use drugs (including women and gender diverse people) experience increased stigma and discrimination, including police encounters, arrests, imprisonment, and child apprehension [19–21]. The intersections of social identities associated with oppression (e.g., racism, misogyny) influence inequities in health and access to health care, resulting in cumulative disadvantage (e.g., poverty, increased health harms such as overdose deaths, HIV/AIDS, and Hepatitis C infections) [22–27].

Working in harm reduction programs (e.g., providing new/sterile equipment, administering naloxone, overdose protocols and/or first aid, and supervising drug consumption) can also have positive health impacts for community harm reduction mobilizers (PWLE) themselves [28, 29]. However, while such initiatives have proven effective in decreasing drug-related illness and death among people who use drugs, recent research has pointed to unintended negative impacts of health models reliant on care provision by those with lived experience of drug use, especially during the overdose death epidemic and in times of austerity and cutbacks [13, 30]. For instance, people with lived experience of drug use working in harm reduction risk overextending themselves beyond employment parameters (e.g., emotional labor, dealing with overdose events outside of work hours) with few systems in place (e.g., employment advocacy, government funding) for support [1, 8, 31]. Further, continued exposure to stigma, workplace discrimination, and/or power imbalances between staff with lived expertise of illicit drug use and staff without, as well as the impact of high stakes employment (e.g., dealing with overdose deaths), can have significant consequences for community harm reduction mobilizers in terms of burn out (e.g., emotional exhaustion), disparity, trauma, and grief.^13^ With growing awareness that the participation of people with lived experience of drug use in harm reduction care can have both health benefits and unintended consequences, their participation in Canada’s overdose response commands further investigation and recognition.

In this paper, we begin to address this gap by examining some benefits and negative aspects of working in harm reduction, drug user unions/groups, and overdose-related interventions across Canada among people with lived experience of drug use. While people with lived experience of drug use are being increasingly engaged in harm reduction and addiction treatment interventions, in many cases without accompanying employee supports, there is a need for continued investigation into the effectiveness of these programs and the impacts on employees. The factors that promote meaningful involvement have not been investigated fully and opportunities to involve those with expertise in drug use in the delivery of substance treatment programming remains under-explored.

## Methods

This study stems from a 5-year project initiative commenced in 2018 that operates as a component of the Canadian research consortium, CRISM (Canadian Research Initiative in Substance Misuse) to address gaps in overdose prevention responses with a leadership working group of people with lived expertise of drug use associated with four regional nodes across Canada: British Columbia; Ontario; Quebec/Atlantic regions; and the Prairies. At the outset, CRISM researchers (without expressed lived experience of illicit drug use) from each regional node invited an average of two people with lived experience of drug use working in harm reduction/drug user unions in their region to join the national working group (later named ‘People with Lived Expertise of Drug Use National Working Group’ by the members). For example, the lead researcher in Vancouver, British Columbia (BC) approached the board of directors for the drug user union, the Vancouver Area Network of Drug Users (VANDU), about the project and asked if they could recommend two people who might join the group. A third member was recommended through PWLE activism networks as someone who could provide rural representation. Membership (e.g., new members) and leadership (e.g., research objectives, meeting facilitation) then emerged directly from the members with lived expertise of drug use, while the lead researcher coordinated the group projects. Membership numbers vary; however, the Acknowledgements section indicates the community harm reduction mobilizers with lived experience of drug use from across Canada who made up the working group when the study was conducted and completed.

Because the group is drawn from people across Canada, meetings occur every 4 weeks via a scheduled conference call. Members of the PWLE National Working Group are compensated for their participation in the group. At meetings, members discuss pertinent issues related to drug use, health and human rights, including ongoing discrimination of people who use drugs, regional (lack of) distribution of harm reduction services, the overdose crisis, structural violence (e.g., gendered violence, poverty, colonialism and systemic racism, drug laws), and the need for more diverse and culturally responsive services. Also discussed is the need to fully recognize the work of people who use drugs as an essential component to health and social services, as well as the hardships, inequity, lack of compensation, and emotional toll of their work. Knowledge sharing among members provides opportunities for identifying and addressing regional differences as well as providing support across regions. During meetings, members also brainstorm about timely research projects that they can collaborate on despite geographical distance, projects that can highlight member experiences, work, and priorities.

Through robust discussion over several months, the group worked to create a short, one-page survey, with survey questions (both closed and open-ended) emerging directly from the meetings to highlight inequities experienced by people who use(d) drugs working in harm reduction. The use of a short survey for data collection fit well with the groups’ time and commitment constraints. This process was informed by community-based research (CBR) practices with structurally marginalized communities [2, 32–34], including CBR protocols developed by people with lived experience of drug use and drug user unions. CBR involves collaborative approaches to conducting research with those most affected in order to further social justice goals [35].

The survey was administered in 2018 at the *National Stimulus: Drugs, Policy and Practice in Canada* conference, as group members and numerous people with lived experience of drug use working in harm reduction or at drug user unions (the recruitment criteria) attended the conference, held in Edmonton, Alberta. The PWLE National Working Group set up a table in a common area to distribute the one-page survey and to inform conference attendees about the group and research project. The lead researcher, project coordinators as well as volunteers from the PWLE National Working Group worked with participants requiring assistance to fill out surveys and to recruit participants, while also attending various conference panels.

The survey included demographic questions, multiple-choice questions (on forms of compensation and issues impacting employment (see Tables 2, 3) as well as a section for respondents to write in their own words about the benefits and the negative aspects of ‘peer’ employment and their recommendations for employment equity for people with lived experience of drug use. This included three open-ended questions: (1) What benefits do you experience as a ‘peer’ working in harm reduction?; (2) What negatives do you experience as a ‘peer’ working in harm reduction?; and (3) What recommendations or changes should be made in your organization to make ‘peer’ work more equitable and/or safe? Surveys took approximately 15 min to complete and participants received a $10 honorarium for their time. Data collection stopped once the conference ended. In total, fifty surveys were completed by people with lived experience of drug use between October 2–5, 2018.

Demographic data were compiled from the surveys into a spread sheet by a research coordinator and analyzed by the working group using Microsoft Excel. All identifiers were removed for the purpose of confidentiality. Written survey responses were thematically analyzed using both deductive (e.g., benefits of working in harm reduction) and inductive (e.g., skill building, tokenism) coding [36]. Thematic codes were then discussed by the working group to ensure that they represented the range of responses by survey participants [35]. The working group then analyzed, reviewed, added to, and commented upon draft documents of this paper, and debated issues significant to critical drug research, including the lack of representation of people of color and the usage of the term ‘peer’ when writing, in relation to terms such as expert, community harm reduction mobilizers, people who use(d) drugs, and people with lived expertise of drug use. The study received ethical approval from the Providence Healthcare/University of British Columbia Research Ethics Board.

## Findings

The survey participants held diverse positions and were engaged in outreach, health system navigation (e.g., advocacy, education, and accompaniment), needle exchange, and overdose prevention work. They held positions as workers with lived experience of drug use, coordinators, supervisors, and management positions. Response rates for all survey questions were high, with an average 95.5% response. Survey participants identifying as white (42%) comprised the largest group, followed by Indigenous (28%), African, Caribbean, Black (6%); East or South Asian (4%); and Bi-racial (4%) (see Table 1). The mean age of survey participants was approximately 44 years of age, and there was a near even distribution of men (48%) and women participants (46%). Additionally, 4% of participants identified as non-binary or androgynous, and 2% of participants identified as two-spirit. The majority (27%) of participants were from British Columbia, specifically Vancouver, while the Prairies (Alberta, Saskatchewan, and Manitoba) produced the second largest proportion of survey participants at 16%. The majority of participants worked in harm-reduction services. While hours of work varied greatly, 42% of participants reported working part-time hours or less, and 40% reported hours that qualified as full-time or greater. The majority of participants were paid by stipend/honorarium (including gift cards) and/or by the hour with only 36% of participants salaried (see Table 2). Survey participants identified *stigma* as the largest issue impacting their work as a person with lived experience of drug use, followed by lack of salary and benefits, and pay inequities between employees with lived experience of drug use and employees not defined by their substance use (see Table 3).

**Table 1:** Participant characteristics

Characteristic	Count (*n* = 50)	%
Mean age	25.5	
Gender		
Men	23	46
Women	24	48
Non-binary or gender diverse	2	4
Two-spirit	1	2
Ethnicity/race		
White	21	42
Indigenous	14	28
African, Caribbean, Black	3	6
East or South Asian	2	4
Other	2	4
No entry	8	16
Node (geographic area)		
British Columbia (Abbotsford, Nanaimo, Nelson, Surrey, Vancouver, Victoria)	27	54
Prairies (Calgary, Edmonton, Winnipeg)	13	26
Ontario (Ottawa, Toronto)	4	8
Quebec/Atlantic (Montreal, Halifax, St. John’s)	6	12
Hours of work		
Part-time or less	20	40
Full-time or more	21	42
Varied greatly	8	16
No entry	1	2

**Table 2:** Forms of compensation

Forms of ‘peer’ payment	Participant responses (*n* = 50)	%
Salaried	18	36
Volunteer	9	18
Stipend/honorarium	20	40
By the hour	19	38

**Table 3:** Issues most impacting employment

Issues that most impact ‘peer’ work	Participant responses (*n* = 50)	%
Stigma	28	56
Health issues	19	38
Unequal work environment	21	42
Pay differences for ‘peers’	26	52
Lack of benefits	26	52
Lack of salary	27	54
Safety	14	28
Trauma (from front line work during the overdose crisis)	25	50
Social assistance (welfare/disability) impacting pay amounts	18	36

Survey participant responses are highlighted below.

### The benefits of ‘peer’ work

Participants identified a range of positive elements attributed to their employment, including personal benefits (building of skills and confidence), community benefits (saving lives, sharing knowledge, and destigmatizing drug use), and relationship building (forming trust).

#### Valued expertise: as a person with lived experience of drug use

In particular, participants described the significance of working as someone with the expertise of lived experience of drug use, as it allowed for shared knowledge, the building of trust, and the forming of meaningful relationships—as the following quotes indicate:

I am working with people I love and nobody knows how we feel and what we need more than those who feel it. (Participant 7, 40-year-old white woman from Vancouver).

Because I lived it, I can relate to people. (Participant 50, 56-year-old Métis man from Edmonton).

Numerous participants stated that their lived experiences and insights were valued by their clients and co-workers, and that being an employee with lived experience of drug use or actively using drugs helped them create compassionate and non-judgmental work environments and brought them insights that would otherwise be absent.

Relationship building is huge. Those who are aware of my status build more trust and state they relate better. (Participant 8, 38-year-old woman from St. Johns, ethnicity not identified).

Participants noted that an additional aspect of being valued for one’s expertise was the increased opportunities to be heard, to have a voice in contexts where drug users’ expertise has historically been denied and/or dismissed.

It wasn’t too long ago that what I had to say was dismissed based by appearance and how I lived my life. Today I have a voice and am able to use it to speak for those who have not found their own voice yet. (Participant 30, 39-year-old South Asian woman from Surrey).

#### Empowerment and confidence-building

Participants explained ways in which learning what people needed, and knowing that they were able to help a community filled with people they genuinely cared about, brought a certain level of fulfillment.

Every ounce of my work provides me with infinite benefits. These benefits are creating and being blessed by the most genuine, raw, meaningful relationships. Witnessing hope and joy in the midst of death and sorrow. Determination, resiliency, and empowerment that I get to be a part of and have my own strengthened alongside. Watching the victories, celebrating even the smallest ones. Coming together in grief; pushing through for change – united. (Participant 1, 28-year-old white woman from Abbotsford).

They described feeling a sense of satisfaction from their work and their ability to link community members to necessary services including harm reduction supplies, daycare services, and social services. Participants commented that having useful knowledge to share served to build confidence:

It boosts my ego knowing that I am doing good by helping another human being. Knowing that my knowledge through lived experience is useful. (Participant 11, 53-year-old white man from Ottawa).

There is nothing better than making a difference in someone’s life. It fills my heart, not my pockets. (Participant 33, 41-year-old woman from Vancouver, ethnicity not identified).

Helping people helps me. (Participant 43, 57-year-old Indigenous man from Edmonton).

#### Knowledge and skill building

Participants reported that their work not only helped them realize their own value, but also allowed them to have a voice amid those who were considered “experts” in the field. Furthermore, the work allowed participants to build upon their own skill set. Communication, negotiation, and life skills were reportedly acquired by participants through their organizational role, along with the opportunity to learn about diverse people. Additionally, participants reportedly enjoyed an increased knowledge of harm reduction strategies as well as increased access to resources (e.g., housing).

I get to interact with all the people on the street. I get to make sure that people have access to clean harm reduction supplies. I get to be in the loop with helping our clients get housing, into detox, or even just to talk. (Participant 2, 38-year-old white woman from Surrey).

The #1 benefit to me is the ability to give back and save lives. (Participant 27, 44-year-old man from Vancouver, ethnicity not identified).

Lived experience was perceived as especially beneficial for community members when employees with lived experience of drug use occupied a position of authority, as expressed in the following quote:

As a peer supervisor, I get to allow the peers working alongside me to use their drug of choice while on shift. The peers working under my supervision are able to have the issues that affect users negatively in other jobs overlooked and not held against them while working in our peer program. (Participant 6, 45-year-old white woman from Vancouver).

Workers who no longer actively used illicit drugs also felt that they could serve as examples to those who were currently using, with one participant stating:

This kind of work I feel is helping me realize my value as a human being in being an example of someone that doesn’t use drugs anymore. Showing that it can be done in the same token I find helps me face my demons head on. Being present with peers and around usage reminds me what I don’t want to be doing in my life anymore and shows me that we have to work hard to remain clean. (Participant 37, 31-year-old man from Nanaimo, ethnicity not identified).

Furthermore, participants reported that as people with lived experience who possessed a range of valuable skills, they had the opportunity to challenge stereotypes and preconceived notions about people who use(d) drugs.

The benefits I experience are disseminated, information about my HIV, HCV, Drug Use, Harm Reduction. Knowing that through [my work] that I saved a life. Just in general [it’s beneficial] for people who have stigma to teach, to not feel like scum, a lowlife. (Participant 11, 53-year-old white man from Ottawa).

I feel like I help people, I help change the status quo, normalize drug use, defend me and my comrades’ rights. (Participant 17, 26-year-old white androgyne from Montreal).

Overall, survey participants described a range of personal benefits generated from working as a person with lived experience of drug use in harm reduction. They also saw the ways in which these benefits extended into the communities in which they belong and work.

### Negative aspects of ‘peer’ work

While the benefits of working in harm reduction were significant, several key concerns arose when survey participants were asked about their negative experiences working as someone with lived expertise of drug use.

#### Tokenism

Tokenism and the devaluing of survey participants’ lived experiences, along with discriminatory attitudes and institutional inequities, were commonly discussed themes. Minimal efforts of inclusiveness and tokenistic engagement were repeatedly identified by people with lived experience of drug use as issues. One participant noted:

Pay me much more. Let me use openly at work. Listen to me. Stop fetishizing/tokenizing me (I don’t speak for all drug users so they should stop asking me). Acknowledge I’m a victim of the drug war and my colleagues are then more privileged (Participant 17, 26-year-old white androgyne from Montreal).

Another participant noted that, while their presence as a PWLE was valued, their expertise was undervalued:

Sometimes I’m not as valued as a “professional” with a Master’s [degree] but lived experience is important too. (Participant 31, 18-year-old Indigenous/Mexican woman from Abbotsford).

Participants noted that identifying as a ‘peer’ can risk overshadowing their expertise:

I don’t often disclose my ‘peerness’ due to a concern of not being taken for what I am. (Participant 8, 38-year-old woman from St. Johns, ethnicity not identified).

One participant succinctly summarized the sentiments of many of the survey respondents and co-authors who work in harm reduction services:

When do I stop being a ‘peer’? (Participant 42, 24-year-old two-spirit Indigiqueer Cree and Ojibwe Femme from Winnipeg).

#### Unrecognized work/expertise and wage inequities

Participants reported that within their organizations they were not taken seriously or as seriously as the academics, ‘experts,’ or other professionals they worked with. Survey participants described feeling as though, despite having valuable insights, expertise, and lived experiences to draw from, they often received patronizing responses from those not defined by their substance use within their organization. Especially concerning for participants was the wage inequity between employees who use(d) illicit drugs and employees that openly did not (including academics, social workers, and service providers). Repeatedly, respondents reported that despite participating in and completing the same type or amount of work, they were paid less (with fewer benefits) than those with more education:

At times, I feel underpaid due to lack of formal education. Some authorities view my work as less than that of my co-workers with degrees. (Participant 46, 27-year-old white woman from Fort Saskatchewan).

Furthermore, some participants described that their role as workers required them to engage in greater amounts of emotional labor that consistently went unrecognized and unpaid, as the following responses illustrate:

low wages, no benefits (Participant 15, 55-year-old Black man from Toronto).

Wage disparity and lack of job security [are negatives to my experiences]. I am the supervisor of the site, the one with all the responsibility. The people I train that work for [the site], and I am their supervisor, make $5/h more than I do. As well I opened [these sites] without a vacation or stress leave and would have no job security if I took a leave but the 3 fulltime [site] staff that began working in our sites [after me] are all on stress leave of 3–5 months securely. (Participant 6, 45-year-old white woman from Vancouver).

Another worker noted that, given the disproportionate hardship many PWLE experience (e.g., discrimination, the targeting of poor and racialized groups by law enforcement), additional support, with attention to the impact of these inequities, was needed from employers:

Meal support. I need understanding and support with transport to work but most employment won’t or can’t support bus fare. It costs me money to start work, clothes, bus fare, food for lunch. (Participant 42, 24-year-old two-spirit Indigiqueer Cree and Ojibwe Femme from Winnipeg).

The required support was envisioned as salaried support with benefits, rather than gift cards and stipends:

My ‘peer’ work is valuable so I should be paid in money, not gift cards or stipends. It should be just as equal as a paid worker [who does not openly use criminalized drugs]. (Participant 11, 53-year-old white man from Ottawa).

It should be noted, however, that for some workers on disability benefits, payment for work by stipends worked well. For many participants, wage discrepancies and a lack of benefits were further evidence of tokenism, as were perceptions that their lived experiences, knowledge, and contributions were not valued as highly as those of co-workers who had attained higher levels of education.

Participants described feeling that despite contributing immense amounts of labor to their organizations and communities, they were not promised the same sorts of security and protections that employees who were not ‘peers’ were able to enjoy (e.g., unemployment benefits or extended health plans).

I need to educate my colleagues all the time (I’m the only peer at [site of employment]). I do more emotional work than my colleagues but [am] paid the same/this invisible workload isn’t recognized/. My job doesn’t end when I leave my job but I’m not paid more. At the beginning, I felt “lower” in the hierarchy at my job. Now it’s better but I’m not “in the group” like everyone else. (Participant 17, 26-year-old white androgyne from Montreal).

Participants noted that the unofficial harm reduction work they engaged in also went unrecognized if they were still active in their substance use. According to participants, providing harm reduction services to their community and ensuring that those who did not access official services were still receiving harm reduction supplies were not considered employment skills and did not qualify a person for any sort of funding or support. This provided further evidence of the ways participants’ work and contributions were devalued.

#### Stigma and discrimination

In addition to tokenistic engagement and wage inequities, participants wrote about the stigmatizing and discriminatory experiences they had while interacting with co-workers who were not defined by their substance use, as well as institutions like the police and social services. Stigma and discrimination could be further compounded for racialized participants, especially Indigenous community members who stated that in addition to discrimination resulting from substance use, they also had to contend with racialized stigma and discrimination. One Indigenous participant described being held to unattainable measures not expected of those who are non-Indigenous:

…As a First Nations Male we experience a lot of co-opting of our narrative (no offense) by academics and [are] held to impossible standards. (Participant 12, 53-year-old Kway-luth man from East Vancouver).

In certain cases, participants described the extent to which stigma and discrimination due to their history of substance use impacted their ability to work:

STIGMA! Even 3 years into my recovery, worked up from the bottom to a paid “professional” position and yet when I walk into meetings w/ the mayor, @ the city, or even @ community meetings w/ executive directors of organizations that serve our community & other service providers, I am often ridiculed or ignored & not even acknowledged. Worst still, is being targeted & having others (esp. “professionals”) call or do other things to try & get me removed from my position, etc. (Participant 1, 28-year-old white woman from Abbotsford).

Regardless of whether participants were active in their drug use or working in a ‘professional’ role, they still had to contend with stigma and discrimination on multiple levels, both at work and outside of work.

The criminalization of people who use drugs further serves to stigmatize and impact the ability of people who use(d) drugs to fulfill their roles as harm reduction workers. For example, one 43-year-old white gender fluid participant from the north coast of BC included not only ‘patronizing responses from non-drug user “experts”’ but also ‘risks of being “outed” to RCMP [Royal Canadian Mounted Police] and MCFD [Ministry of Children and Family Development] in my community’ as significant barriers to working in harm reduction. Participants further cited active dismissal by law enforcement and medical professionals as a problematic aspect of their work:

Where I live, the Vancouver police don’t give a care in the world. A lot of hospitals just push people away. It’s like they want us not to bother them, because I’ve seen a lot of things happen. Also, sometimes we get attitude from the people out there suffering. (Participant 34, 50-year-old Indigenous woman from Vancouver).

Participants described the impact of compounding discriminatory practices by ‘experts,’ hospital staff, healthcare and service providers, as well as law enforcement:

Tokenism. Exploitation. Paternalism. “Seen as threat.” Criminalized. Devalued by some. (Participant 28, 43-year-old white woman from Abbotsford).

### Recommendations for change

Participants’ recommendations for improved work spaces focused on increasing equity and capacity building and a call for organizational restructuring that promoted equitable, anti-racist feminist, and transparent practices and policies. Changes that addressed tokenization and devaluing of people who use(d) drugs and their experiences were of the utmost concern for participants. Equal pay for equal work was one of the most recurrent recommendations, along with employment health benefits and resources, such as extended health care and leadership training, that would support people with lived experience who were working for substance use and harm reduction organizations.

Not only do organizations need to recognize the cost that people who use(d) drugs incur simply attempting to fulfill their work obligations, but factors that facilitate uncomfortable and exclusionary work environments must also be addressed. Addressing power imbalances was recommended as a solution to inequitable work environments, with some participants asking for ‘more leadership roles’ for people who use(d) drugs and also for those systemically marginalized, such as Indigenous employees:

[We need] to make the system set up more available for peer support and aboriginal individuals to be able to be managers and our team leads and to be treated with respect and valued for their lived experience and to be viewed as just as important. (Participant 44, 32-year-old Indigenous woman from Edmonton).

One 31-year-old man from Nanaimo usefully suggested that organizations,

… run on a model that lets everyone get a chance to facilitate, schedule, ‘run the show,’ so it can empower the peers who use [drugs] and show they are of value to the team as they are more than a connection to the streets.

Additionally, a 38-year-old Misipawistik First Nations woman recommended that organizations pair coordinators with and without lived experience of illicit drug use together so that they could,

…work together with peers as equals learning and growing together. Why set up people for failure?

A few participants expressed the view that people with lived experience of drug use should not be able to come to work under the influence. However, the majority of participants maintained that they should be allowed to use drugs openly without being stigmatized or discriminated against, and should also be allowed to use the services at their own organizations provided for people who use drugs (such as a safe injection room). Furthermore, an increase in community services was heavily advocated for by participants who recommended supports such as retractable needles, increased access to beds and detoxes, and increased hours for services. In particular, participants emphasized the need for increased support for those living on the streets:

More for the people out on the streets to strongly push for more help from others and ask people what they really want to do. Some people should be out there at all times where ever they are using [drugs]. (Participant 34, 50-year-old Indigenous woman from Vancouver).

Some participants, however, expressed frustration that services were often only accessible to those most destitute, those who were currently living on the streets, while there was an absence of preventative care. Further, a difficult concern expressed by participants was community level hostility toward employees with lived experience of drug use, which could take the form of harassment and sometimes violence. They noted that some community members seemed to believe that people who use(d) drugs and worked in organizations or held positions on advisory boards felt that they were better than those who use(d) drugs and utilized the agencies’ services. Participants themselves expressed confusion over their status, and whether they were still ‘peers’ if they were no longer actively using drugs. As previously stated, the term ‘peer’ itself was identified in the survey as an issue, and some participants suggested replacing the word with ‘experts.’

## Discussion

The benefits participants attributed to their employment, including the building of skills, expertise, confidence, knowledge sharing, and most significantly, saving lives, served to enable a sense of decreasing the stigmatization of drug use, and the building of confidence and community-based trust among participants and their clients. However, participants’ experiences of discriminatory attitudes and institutional and wage-based inequities, resulted in what was described as feelings of limited inclusiveness, tokenistic engagement, unrecognized labor and expertise and a sense of thwarted ability for career advancement as a person with lived experience of drug use working in harm reduction.

Qualitative research on ‘peer’ employment in harm reduction services, including overdose prevention and response, reveal similar benefits, in particular the effectiveness of peer-led models of care. In reaching vulnerable communities, however, they also detail the particular struggles experienced in their efforts to care for others who use illicit drugs, including material and pay inequities that factor into negotiation and navigation of the invisible boundaries that demark what constitutes a ‘peer,’ and frought workplace policies regarding drug use/abstinence, as described by our study participants, and which remain unresolved [1, 4, 9, 28, 37, 38]. Significantly, the involvement of people with lived experience of drug use has also resulted in increased diversity within many of these service settings, with socially and economically marginalized women and Indigenous people who use(d) drugs taking a particularly prominent role in emerging programming [31, 39–42]. This has potential benefits that have not been adequately examined, as most work on these approaches—amid the overdose epidemic and otherwise—has homogenized the roles of people who use(d) drugs rather than more broadly considering their diversity; a consideration highlighted by some survey participants that warrants further exploration.

Participant comments emphasize that meaningful involvement of people who use drugs working in harm reduction requires attention to the intersecting oppressions that impede their participation. Critical scholars note that poverty and economic marginalization shape the lives of many people who use criminalized drugs [15, 18, 43–45], including those working in harm reduction services. Due to ongoing colonialism and the legacy of slavery for Indigenous and Black people who use criminalized drugs, racial profiling, discrimination, arrest, and imprisonment are heightened [20, 21, 44]. Thus, Indigenous and Black people are overrepresented in Canadian provincial jails and federal prisons [21]. Indigenous women and girls continue to be subject to gendered violence, as the inquiries on missing women and girls have made clear [46, 47]. Indigenous and Black families are also profiled by child protection services, and families are often torn apart [48]. Due to the ongoing legacy of colonialism, Indigenous peoples and low income/rural communities in Canada have been disproportionately affected by the overdose crisis [22]. For those people and communities most marginalized in Canada, the impact of structural violence, including punitive drug laws and policies, shapes daily life, including employment. As one participant stated:

I really feel beat down with the opioid crisis and also I get a little beat up and frustrated with the policies and laws. (Participant 29, 43-year-old Métis man from Victoria).

Such intersecting oppressions impede the work capacity of already stigmatized populations who use drugs. Continued exposure to stigma, social, legal and workplace discrimination, and/or power imbalances, combined with the impact of an overdose crisis and high stakes employment (e.g., dealing with overdose-related deaths at and outside of work), can have significant consequences for community harm reduction mobilizers, including emotional exhaustion. Just as early drug user ‘unions’ emerged to challenge punitive drug policies in and outside of Canada [49, 50], collective organizing and the unionization of harm reduction workers has been one avenue advocated by some people with lived experience of drug use as a means to achieve equity toward a non-hierarchical workforce that is attentive to labor rights [51]. Additional policy recommendations include large-scale structural changes that address inequities of hierarchical ‘peer’ employment for PWLE, including increased leadership roles for diverse PWLE, pay equity and benefits, as well as more supportive working environments attentive to the intersecting social-structural factors (poverty, criminalization, racism, gendered violence) impacting the everyday lives of PWLE working in harm reduction.

This study has several limitations. Despite attempts to recruit and include a diverse range of PWLE, participants’ views may not be reflective of all ‘peer’ experiences working in harm reduction in and outside of Canada, as our data were gathered at the site of one specific harm reduction conference in Alberta, Canada. Furthermore, British Columbia residents made up the majority of participant responses, creating a potential geographic bias. Additionally, limited gender and racial diversity among study participants restricted our ability to fully explore specific ‘peer’ experiences of working in harm reduction, through an intersectional lens. Finally, qualitative surveys are limiting when compared to the dialogue inherent to qualitative interviews, which can elicit more fulsome and nuanced insight into participant perspectives. However, the use of a short survey for data collection (which is less time consuming), provided a useful method to gather some pertinent issues experienced by PWLE across Canada, without overburdening the national working group members attending the conference.

## Conclusion

Harm reduction programs in Canada continue to struggle to provide services against the backdrop of federal, provincial, and municipal cut-backs in social supports and funding for health, housing, education, and harm reduction. These cut-backs over many decades have left front-line workers, including people with lived experience of drug use working in harm reduction, bearing the brunt of government austerity and neglect. It is our hope that our findings will inform structural change, government funding, and equitable workplace policies for people with lived experience and expertise of drug use working in harm reduction services. Our findings contribute to a growing body of work by and for people with lived experience of drug use working in harm reduction. In order to fully understand harm reduction work, further research by and for people with lived experience is essential.

## Data Availability

Not applicable.

## Abbreviations

PWLE: People with lived experience/expertise of drug use
BC: British Columbia
RCMP: Royal Canadian Mounted Police
MCFD: Ministry of Children and Family Development
